# Analysis of the TGFβ-induced program in primary airway epithelial cells shows essential role of NF-κB/RelA signaling network in type II epithelial mesenchymal transition

**DOI:** 10.1186/s12864-015-1707-x

**Published:** 2015-07-18

**Authors:** Bing Tian, Xueling Li, Mridul Kalita, Steven G. Widen, Jun Yang, Suresh K. Bhavnani, Bryant Dang, Andrzej Kudlicki, Mala Sinha, Fanping Kong, Thomas G. Wood, Bruce A. Luxon, Allan R. Brasier

**Affiliations:** Department of Internal Medicine, University of Texas Medical Branch (UTMB), Galveston, TX USA; Sealy Center for Molecular Medicine, UTMB, Galveston, TX USA; Institute for Translational Sciences, UTMB, Galveston, TX USA; Department of Biochemistry and Molecular Biology, UTMB, Galveston, TX USA; Bioinformatics Program, UTMB, Galveston, TX USA

**Keywords:** Epithelial mesenchymal transition, Transforming growth factor β, Nuclear factor κB, RNA-Seq, Generalized linear modeling, Transcription factor enrichment

## Abstract

**Background:**

The airway epithelial cell plays a central role in coordinating the pulmonary response to injury and inflammation. Here, transforming growth factor-β (TGFβ) activates gene expression programs to induce stem cell-like properties, inhibit expression of differentiated epithelial adhesion proteins and express mesenchymal contractile proteins. This process is known as epithelial mesenchymal transition (EMT); although much is known about the role of EMT in cellular metastasis in an oncogene-transformed cell, less is known about Type II EMT, that occurring in normal epithelial cells. In this study, we applied next generation sequencing (RNA-Seq) in primary human airway epithelial cells to understand the gene program controlling Type II EMT and how cytokine-induced inflammation modifies it.

**Results:**

Generalized linear modeling was performed on a two-factor RNA-Seq experiment of 6 treatments of telomerase immortalized human small airway epithelial cells (3 replicates). Using a stringent cut-off, we identified 3,478 differentially expressed genes (DEGs) in response to EMT. Unbiased transcription factor enrichment analysis identified three clusters of EMT regulators, one including SMADs/TP63 and another NF-κB/RelA. Surprisingly, we also observed 527 of the EMT DEGs were also regulated by the TNF-NF-κB/RelA pathway. This Type II EMT program was compared to Type III EMT in TGFβ stimulated A549 alveolar lung cancer cells, revealing significant functional differences. Moreover, we observe that Type II EMT modifies the outcome of the TNF program, reducing IFN signaling and enhancing integrin signaling. We confirmed experimentally that TGFβ-induced the NF-κB/RelA pathway by observing a 2-fold change in NF-κB/RelA nuclear translocation. A small molecule IKK inhibitor blocked TGFβ-induced core transcription factor (SNAIL1, ZEB1 and Twist1) and mesenchymal gene (FN1 and VIM) expression.

**Conclusions:**

These data indicate that NF-κB/RelA controls a SMAD-independent gene network whose regulation is required for initiation of Type II EMT. Type II EMT dramatically affects the induction and kinetics of TNF-dependent gene networks.

**Electronic supplementary material:**

The online version of this article (doi:10.1186/s12864-015-1707-x) contains supplementary material, which is available to authorized users.

## Background

The airway mucosa consists of highly polarized, differentiated epithelial cell types, whose primary role is to restrict fluid loss and limit inhaled particulate access to the internal milieu [1]. Exposure to aero-allergens, respiratory viruses or reactive oxygen stress, induces anchorage-dependent cell death (anoikis). During the process of anoikis, epithelial sloughing and disruption of the basement membrane releases sequestered extracellular matrix-associated epithelial growth factors, transforming growth factor (TGF), epidermal growth factor (EGF) and fibroblast growth factor (FGF). These epithelial growth factors activate gene expression programs in resident stem cell population (basal cells) to initiate epithelial repair and regeneration.

TGFβ-stimulation of normal epithelial cells activates a de-differentiation program to promote cellular regeneration and extracellular matrix formation [1–3], referred to as Type II epithelial mesenchymal transition (EMT; [4]). Type II EMT induces loss of apical-basal polarity through cytoskeletal reorganization and dissolution of tight junctions. Concomitantly the cells express mesenchymal smooth muscle cell actin and intermediate filament vimentin (VIM) enhancing motility, and secrete collagen (Col1A), fibronectin (FN1), and matrix metalloproteinases (MMPs) promoting extracellular matrix deposition. Collectively this response promotes airway remodeling with expansion of the smooth muscle cell layer, repair of the epithelial surface and fibrosis [4, 5]. In this way, the airway epithelium plays a primary role in the resolution of injury or, if unresolved, the pathogenesis of chronic airway disease [6].

EMT is a dynamic and reversible epigenetic reprogramming event that plays a role in embryogenesis, organ homeostasis in response to acute injury, or cancer progression/metastasis, referred to as Types I, II, or III EMT, respectively [4]. Using Type III EMT as a model of cancer progression and metastases, the molecular signaling of how TGFβ1 initiates EMT has been studied extensively. Ligand induced activation of the transmembrane serine/threonine kinase TGFβ receptor type II (TGFβRII) recruits and phosphorylates TGFβRI to signal through Smad-dependent, “canonical” and Smad-independent “noncanonical” pathways [7]. In the canonical pathway, phosphorylated Smad2/3 binds to Smad4 and the complex then translocates to the nucleus. The noncanonical signaling pathways involve downstream PI3K/Akt, Ras small GTPases, Wnt/β-catenin, ERK, p38, and JNK. Collectively, the canonical and noncanonical pathways converge on a core set of transcription factors that function to initiate and maintain EMT: SNAIL (SNAI) 1/2, zinc finger E-box binding (ZEB) 1/2, Twist 1/2, Goosecoid and others [8].

The core EMT transcription factors mediate a coordinated series of gene activation/repression and chromatin reprogramming events to shift cells to the mesenchymal phenotype. The activated Smad 2/3/4 trimer binds to Smad-binding elements in the regulatory regions of *SNAIL*, *JunB* and *c-Jun*, activating their expression by recruiting coactivators including the cAMP-response element binding protein (CBP)/p300 histone acetyltransferases [9]. SNAIL, in turn, represses *ECad* and zona occludin-1 genes by recruiting the polycomb complex, producing silencing histone modifications [10–12]. Smad signaling also increases expression of *ZEB1/2*, resulting in *MMP* and *collagen 1A* expression [8]. ZEB interacts with lysine-specific demethylase (LSD1), a protein involved in histone demethylation and chromatin reprogramming in EMT [13, 14]. Together these proteins coordinate both the repression of epithelial related genes and activation of mesenchymal genes.

Because of the temporal interplay of diverse signaling programs required to initiate and maintain EMT reprogramming, the EMT is highly modified by the state of cellular transformation and concomitant activation of extracellular signaling pathways. Oncogenic mutations in K-ras, activation of Wnt signaling, ROS stress and activation of insulin-like growth factor pathways that cross-talk with the TGFβ pathway modify the expression of the EMT program [15]. As a result, the EMT program can be modulated by extracellular matrix interactions [16], and, of interest here, pro-inflammatory monocyte derived cytokines. TNF is a prototypical monokine [16, 17], whose actions trigger activation of p38 MAPK and JNK, essential components of the noncanonical TGFβ signaling pathways [18, 19], and induce EMT in K-ras transformed epithelial cells through the actions of NF-κB on the Twist EMT core transcription factor [16, 20]. However, the role of NF-κB signaling in the EMT of normal epithelial cells is not known.

In this study we sought to examine the gene program of Type II EMT and to identify how this process was modulated by interaction with the innate signaling pathway. A well-established model of TGFβ-induced EMT was applied to primary immortalized human small airway epithelial cells (hSAECs) to identify the gene expression networks responsible [5], and understand how activation of the innate response was modulated by EMT. Surprisingly, we observed that TGFβ produced a gene expression program that was significantly enriched in NF-κB-dependent genes identified by comparison to TNF dependent genes and to RelA enriched target genes in public ChIP-Seq data. Moreover, Type II EMT produces profound rewiring of the TNF gene program, skewing the pathway towards expression of integrin signaling to maintain the EMT state. We demonstrate that inhibiting NF-κB/RelA via gene silencing or by inhibition of the IKK regulatory kinase blocked TGFβ-induced EMT. These data indicate that NF-κB/RelA gene expression program is a major regulator of TGFβ-induced Type II EMT.

## Methods

### hSAEC culture and EMT transformation

An immortalized human small airway epithelial cell (hSAEC) line was established by infecting primary hSAECs with human telomerase (hTERT) and cyclin dependent kinase (CDK)-4 retrovirus constructs [21]. The immortalized hSAECs were grown in SAGM small airway epithelial cell growth medium (Lonza, Walkersville, MD) in a humidified atmosphere of 5 % CO_2_. For induction of EMT, hSAECs were TGFβ stimulated for 15 days (10 ng/ml, PeproTech, Rocky Hill, NJ). The small molecule inhibitor of IKK, BMS345541 was purchased from Sigma Aldrich and used at 10 μM [22].

### Fluorescence microscopy

hSAECs were incubated in the absence or presence of TGFβ (10 ng/mL) for 15 days, re-plated on glass cover slips pretreated with rat tail collagen (Roche Applied Sciences) and fixed with 4 % paraformaldehyde in PBS. Afterwards, the fixed cells were stained with Alexa Fluor® 568 phalloidin for cytoplasmic distribution of F-actin (shown in red color) and also counterstained with 4’, 6-diamidino-2-phenylindole (DAPI) for nuclear staining (shown in blue color). The cells were visualized with a Nikon fluorescence confocal microscope at a magnification of 63× [5, 23].

### Quantitative real time PCR (Q-RT-PCR)

Reverse transcription was performed on 1 μg of total RNA with random primers, utilizing SuperScript III first-strand synthesis system for RT-PCR (Life technologies, Invitrogen) under conditions recommended by the manufacturer. Equivalent amounts of RNA were assayed by quanitative (Q) Q-PCR. The PCR reaction consisted of SYBR Green® PCR Master Mix (Bio-Rad), template cDNA and assay primers (Table 1) in a total reaction volume of 20 μl. Thermal cycling (50 °C, 2 min; 95 °C, 10 min; and 40 cycles at 95 °C, 15 S; 60 °C, 1 min) was performed using a MyiQ Single Color Real-Time PCR Detection System (Bio-Rad). Threshold cycle numbers (Ct) were defined as fluorescence values, generated by SYBR green binding to double stranded PCR products, exceeding baseline. Relative transcript levels were quantified as a comparison of measured Ct values for each reaction [24], normalized using cyclophilin as an internal control.

**Table 1:** PCR Primers for genes in Q-RT-PCR analysis

	Sequence (5’- 3’)	
Primer Set	Forward	Reverse
hCol1A	CCAGAAGAACTGGTACATCAGCA	CGCCATACTCGAACTGGAATC
hFN1	CGGTGGCTGTCAGTCAAAG	AAACCTCGGCTTCCTCCATAA
hIL-6	CTGGATTCAATGAGGAGACTTGC	TCAAATCTGTTCTGGAGGTACTCTAGG
hSNAIL1	GCGCTCTTTCCTCGTCAGG	GGGCTGCTGGAAGGTAAACTCT
hTwist1	TCTCGGTCTGGAGGATGGA	CAATGACATCTAGGTCTCCG
hVIM	GCTCAATGTTAAGATGGCCCTT	TGGAAGAGGCAGAGAAATCCTG
hZEB1	GATGATGAATGCGAGTCAGATGC	ACAGCAGTGTCTTGTTGTTGT
hPPIA	CCCACCGTGTTCTTCGACATT	GGACCCGTATGCTTTAGGATGA
mIL-6	TAGTCCTTCCTACCCCAATTTCC	TTGGTCCTTAGCCACTCCTTC
mSNAIL1	CACACGCTGCCTTGTGTCT	GGTCAGCAAAAGCACGGTT
mZEB1	ACCGCCGTCATTTATCCTGAG	CATCTGGTGTTCCGTTTTCATCA
mPPIA	GAGCTGTTTGCAGACAAAGTTC	CCCTGGCACATGAATCCTGG

### RNA extraction and qualification

Total cellular RNA was extracted using either RNAqueous™ phenol-free total RNA isolation kits (Life Technologies, CA) or Quick-RNA MiniPrep kits (ZYMO Research) according to the manufacture’s recommendations. RNA was quantified spectrophotometrically using a NanoDrop ND-1000 (NanoDrop Technologies, DE). Quality of the purified RNA was assessed by visualization of 18S and 28S RNA bands using an Agilent BioAnalyzer 2100 (Agilent Technologies, CA). The resulting electropherograms were used in the calculation of the 28S/18S ratio and the RNA Integrity Number.

### Library construction and sequencing

Poly-A+ RNA was selected from total RNA (1 μg) using oligo dT-attached magnetic beads. Bound RNA was fragmented by incubation at 94 °C for eight (8) minutes in 19.5 μl of fragmentation buffer (Illumina). First and second strand synthesis, adapter ligation and amplification of the library were performed using the Illumina TruSeq RNA Sample Preparation kit under conditions prescribed by the manufacturer (Illumina). Samples were tracked using “index tags” incorporated into the adapters. Library quality was evaluated using an Agilent DNA-1000 chip on an Agilent 2100 Bioanalyzer. Quantification of library DNA templates was performed using qPCR and a known-size reference standard. Cluster formation of the library DNA templates was performed using the TruSeq PE Cluster Kit v3 (Illumina) and the Illumina cBot workstation using conditions recommended by the manufacturer. Template input was adjusted to obtain a cluster density of 700–900 K/mm2. Paired end 50 base sequencing by synthesis was performed using TruSeq SBS kit v3 (Illumina) on an Illumina HiSeq 1000 using protocols defined by the manufacturer.

### GLM modeling and pathway analysis

As shown in Table 2, the experimental design has six sample groups and requires a two factor analysis for time of TNF stimulation (“Time”) and presence of TGFβ stimulation (“Transformed”). For this purpose we employed the Generalized Linear Modeling (GLM) capabilities in the R Bioconductor package edgeR [25] to perform the data modeling phase of the analysis within our data analysis pipeline shown in Fig. 2b. EdgeR was developed to analyze differential count data arising from designed experiments such as RNA-Seq with single and multiple experimental factors and small numbers of replicates (here n = 3). It includes important statistical methods such as scaled normalization using trimmed means, the negative binomial distribution to describe read count variability, estimates of gene specific dispersion parameters by conditional maximum likelihood using Empirical Bayes methods, exact tests for differential gene expression analysis (DEGA) of one factor experimental designs and GLM for DEGA on multiple factor designs.

**Table 2:** Abbreviations and descriptions of the sample groups for differential gene expression (DGE) analysis

Code	Description
CE	Control (C) untransformed Epithelial cells (E)
CM	Control (C) TGFβ transformed Mesenchymal cells (M)
T1E	TNFα treated (T) E cells 1 h post treatment
T1M	TNFα treated (T) M cells 1 h post treatment
T12E	TNFα treated (T) E cells 12 h post treatment
T12M	TNFα treated (T) M cells 12 h post treatment

The raw NGS analysis passed eleven separate quality analyses by FastQC [26]. Reads were aligned using TopHat2 [27], a fast splice junction mapper that aligns RNA-Seq reads using the ultra-high-throughput short read aligner Bowtie2 [28], using the Burrows-Wheeler index method. The alignment across all 18 samples produced a mean Overall Read Alignment Rate of 96.4 ± 0.4 % and a mean Concordant Pair Alignment Rate of 88.0 ± 0.2 %, confirming that the read alignment was excellent and that data was of high quality. (The results of QA/QC of RNA Seq are provided at Additional file 1).

As shown in Fig. 2b, edgeR used the resulting RNA-Seq read count data to build the counts table in edgeR to begin DGEA. Our experimental design has six groups of cell types (Table 2) each comprised of three samples, thereby giving a total of 18 sample columns with 23,710 rows of read counts, each row representing a unique gene in the count table.

Data filtering by removing unresponsive (i.e., uninformative) genes, was performed to improve the power of the study by reducing effects of multiple testing correction. We use this step to eliminate seemingly unresponsive genes (rows) from the count table using our rule that to be considered responsive, at least one of the six groups has to have a significant number of counts in at least two of its three samples. This filtering step culled out 7,346 rows resulting in a final read count table having 16,364 genes with a mean library size of 4.54E^07 across the 18 samples. This reduced read count table resulted in a mean scaling factor of 1.002 (range: 0.7985 – 1.0990).

Before creating a model design, we identified the model factors (Transformed. Time) and levels to edgeR as [N.0 h, N.1 h, N.12 h, Y.0 h, Y.1 h, Y.12 h] where N = no TGFβ transformation, Y = transformation with TGFβ and hr = time post TNFα treatment in hours. From these factors and levels we can identify the important contrasts that we would like to explore in our DEGA as shown in Table 3. We then designed the statistical model that was most appropriate for guiding the analysis in edgeR. This approach allowed us to optimize the information content while minimizing type I and type II errors, so we can then select those genes most suitable for pathways analysis.

**Table 3:** Compared sample group pairs (contrasts) in the DGE analysis

Contrasts
CM–CE
T1E–CE
T12E–CE
T12E–T1E
T1M–T1E
T12M–T12E
T1M–CM
T12M–CM
T12M–T1M

The edgeR GLMs are non-linear models requiring iterative fitting and dispersion estimation to allow it to account for variations in gene abundance between RNA samples. EdgeR uses the Cox-Reid profile-adjusted likelihood method in estimating dispersions and the read count data is modeled using the negative binomial distribution because it accounts for overdispersion, which the Poisson model does not. This is important in RNA-Seq experiments because the observed variance (i.e., dispersion) in the read count data is often greater than that predicted from statistical theory. The tagwise dispersion was estimated in a gene-wise fashion in three steps using empirical Bayes shrinkage via a weighted likelihood method. Our estimated common dispersion was 0.01548079 which suggests that our sequencing quality is very good.

We next performed the GLM fit using the statistical design then performed the GLM Likelihood Ratio Test (LRT) for each of the contrasts in Table 3. Using each of the LRT outputs, we then performed Bonferroni FWER [29] and Benjamini and Hochberg FDR [30] multiple testing corrections on the DGE for each contrast using the edgeR topTags method. For further analysis such as pathway analysis, gene selection stringency is a key issue so we used a 2× absolute fold change cutoff before p-value filtering. This was a very strong response field and was amenable to a very high level of stringency so we focused on the Bonferonni corrected results with a FWER p-value cutoff of 0.00001 to insure that genes chosen for further analysis were reliably responsive to our experimental conditions and to keep the number of genes in the downstream analytical pool reasonable. Differentially expressed genes are shown in Additional files 2, 3, 4, 5, 6, 7, 8, and 9.

Venn diagrams were created to examine the intersections and relative complements of groups of contrasts (treatment pairs compared) that were deemed biologically interesting using locally developed tools. Subsets of genes identified as interesting were explored using QIAGEN’s Ingenuity® Pathways Analysis suite (IPA®, QIAGEN Redwood City, www.qiagen.com/ingenuity) for pathways, networks, and functional analyses.

### Transcription factor enrichment analysis

ChIP-X data were downloaded from the website http://amp.pharm.mssm.edu/lib/chea.jsp in April 2014. The ChIP-X data consist of 148 transcription factors (TFs) and their respective target genes (TGs) derived from ChIP-chip, ChIP-Seq, ChIP-PET or DamID experiments in 237 publications [31]. Since TFs in different cell lines and treatments may result in different TF binding data, we separated experiments on the same TF, resulting in 345 combinations of TF and experiments performed on human, mouse and rat cells. Among the 345 TF binding experiments, 148 were performed on human. Here we refer to the ChIP-X data as the ChEA ChIP-X data set.

We also downloaded the uniform histone and TF binding peaks from the Encyclopedia of DNA Elements (ENCODE) Consortium website (http://ftp.ebi.ac.uk/pub/databases/ensembl/encode/integration_data_jan2011/byDataType/signal/jan2011/bigwig/) to serve as a second data set. As described above, we separated binding peaks of the same TF from different experiments, which results in 1,169 distinct ChIP-Seq experiments. The target genes corresponding to the TF binding peaks were identified according to PAVIS criteria by using our own scripts [32]. Here the coordinates of the peaks were mapped based on the hg19 refFlat.txt file downloaded from UCSC on Feb. 2012.

Differentially expressed genes (DEGs) between epithelial cells and mesenchymal cells were identified with FPKM fold change ratio > = 2 or < = − 2 and q-value < =0.01 from the RNA-Seq data. 3,487 differentially expressed genes were identified among the 23,284 genome-wide genes considered. Among the DEGs, 2010 genes are downregulated and 1477 are upregulated.

To determine if the transcriptional program regulated by a specific transcription factor from the above 148 transcription factors was activated in the EMT process or not, the hypergeometric probability distribution was used as the background distribution. The enrichment for each transcription factor regulated target gene (TG) was calculated by p-value = 1-hygecdf (k-1, N, K, n) using the Matlab build-in function hygecdf. The number of overlapping genes between the DEGs and the target genes for the specific transcription factor is k; N is the number of genome-wide genes: 23,284; K, the number of the TGs of the TF in the ChIP-data; n, the number of DEGs. For multiple testing corrections, Bonferroni FWER was then performed by multiplying the p-value with the number of TF binding experiments, i.e., 345 TF and experimental combinations used in the analysis based on the ChEA ChIP-X data and 1,169 that based on ENCODE ChIP-Seq data as the corrected p-value. Enrichment fold ratio was calculated by k/n/(K/N). If the corrected p-value by Bonferroni correction was smaller than 0.01 and fold ratio is greater than 1.5, the corresponding transcription factor was considered significantly enriched. For the TF enrichment analysis on ENCODE ChIP-Seq data, a Benjamini-Hochberg correction was also performed to estimate the false discovery rate (FDR) and q-value.

### Hierarchical clustering analysis

TF and target genes were subjected to hierarchical clustering analysis using the Euclidean distance measure and the Ward. D2 linkage function in Spotfire (TIBCO). Variance, Skewness, and Kurtosis of dendrogram results were compared to 1000 random permutations of the data.

### Comparison of type II and type III EMT programs

We compared DEGs in Type II EMT with a publicly available dataset from a model of Type III EMT (GSE177708, NCBI Gene Expression Omnibus). Affymetrix microarrays were used to measure gene expression in human adenocarcinoma cell line A549 after TGFβ induction of EMT. Genes in the two datasets were matched by Entrez ID, and only genes that were common to both datasets, and detected to be expressed in the microarray, were kept for further analysis.

## Results

### Induction of the EMT program

**Fig. 1:**
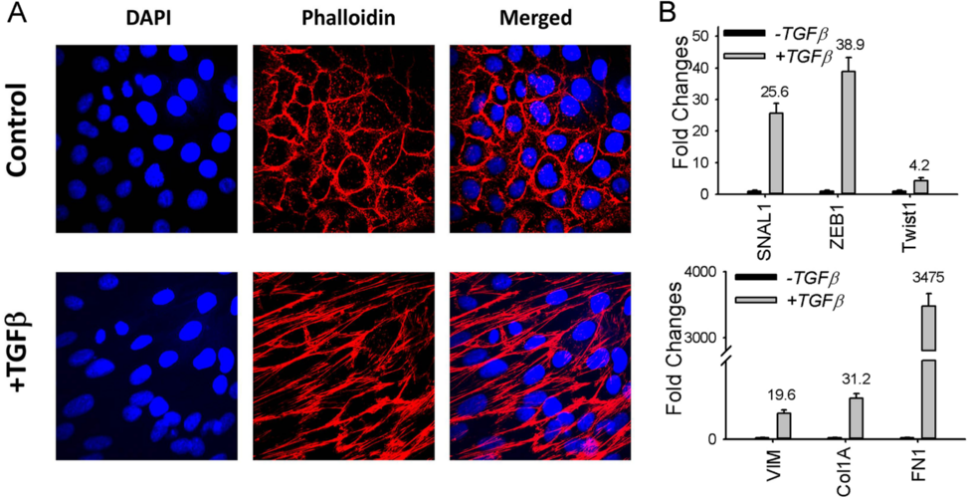
Induction of Type II EMT. **a** Primary human small airway epithelial cells (hSAECs) were incubated in the absence or presence of TGFβ (10 ng/mL) for 15 days. Cells were fixed, stained with Alexa568-conjugated phalloidin (for distribution of F-actin, shown in red color) and DAPI (a nuclear DNA stain, shown in blue color), and examined by confocal microscopy. **b** Total RNA was extracted, purified, and reverse-transcribed. The expression of core transcription factors (*SNAIL1, Twist1*, and *ZEB1*, upper panel) and mesenchymal markers (Vimentin (*VIM*), collagen 1A (*Col1A*), and fibronectin (*FN1*), lower panel) were examined by Q-RT-PCR. Shown is fold-change mRNA abundance normalized to cyclophilin. Data are from three independent experiments

We have previously validated a model of Type II EMT using a continuously replicating line of human small airway epithelial cells (hSAECs) immortalized with human telomerase (hTERT) and CDK4 expression [21]. These cells show a stable epithelial morphology and differentiated cytokeratin isoforms after over 100 population doublings, express the stem cell marker p63 and high levels of p16INK4a, and have an intact p53 checkpoint pathway [21]. In the absence of TGFβ stimulation, hSAECs assumed a normal cuboidal morphology with perinuclear cytoplasmic distribution of F-actin, detected by fluorescence microscopy using confocal microscopy after staining with Alexa568-conjugated phalloidin (Fig. 1a). In response to chronic TGFβ stimulation, the cells acquired an elongated shape with markedly induced F-actin staining. This morphological change of enhanced front-rear polarity and actin rearrangement into cytosolic stress fibers are characteristic morphological changes of EMT [5, 33].

To demonstrate the induction of the Type II EMT gene program, the steady state expression of mesenchymal genes and EMT-associated transcription factors were assessed by Q-RT-PCR. TGFβ-treatment induced the expression of *FN1*, *Col1A* and *VIM* by 3,475-, 31- and 20-fold, respectively (Fig. 1b). Similarly, the EMT core transcription factors *SNAIL1*, *ZEB1* and *Twist1* were induced by 26-, 39- and 4-fold, respectively (Fig. 1b). Together these data suggest that TGFβ induces morphological and gene signatures of stable Type II EMT in hSAECs.

### Analysis of the EMT gene program

**Fig. 2:**
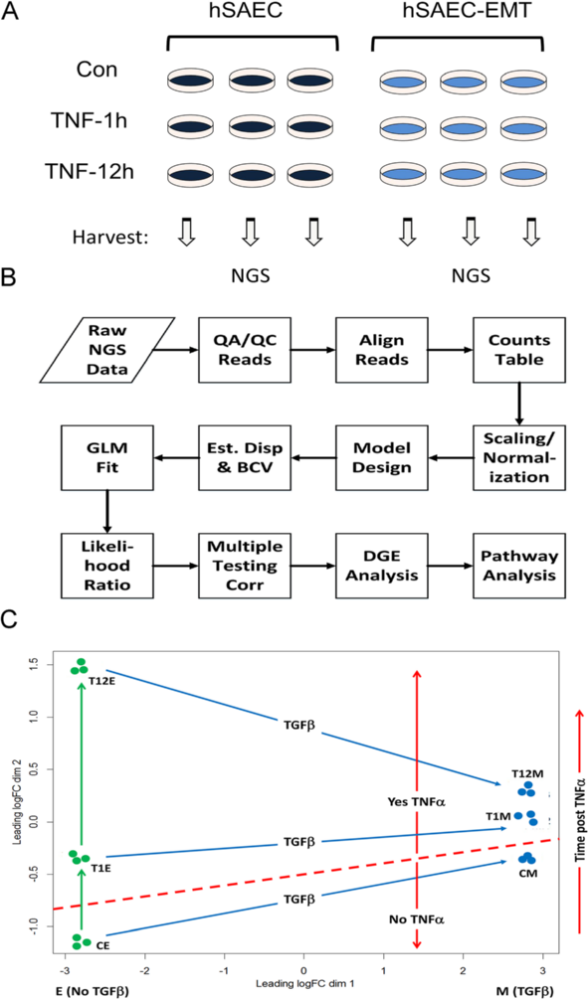
Experimental perturbation and RNA-Seq analysis. **a** Experimental design. Triplicate biological replicates were subjected to no treatment (hSAEC) or TGFβ treatment (hSAEC-EMT). Cells were stimulated with TNFα for 0, 1 or 12 h prior to RNA extraction. **b** Flow diagram for RNA-Seq GLM data analysis. Abbreviations: NGS, next generation sequencing; QA/QC, quality assurance/quality control; GLM, generalized linear model; DGE, differential gene expression. **c** Multidimensional unsupervised clustering analysis of RNA-Seq data sets. Shown is the similarity of each replicate based on TGF treatment or time post TNF and their clustering by group. Abbreviations: CE, control (unstimulated) epithelial cells; CM, control mesenchymal cells

To understand the core EMT program and how TNF signaling modulates its expression, hSAECs were subjected to TGFβ induced reprogramming, and stimulated in the absence or presence of TNF for 1 or 12 h. Each experimental condition was reproduced in triplicate, representing 18 samples subjected to RNA-Seq analysis (Fig. 2a). The schema used in RNA sequence analysis is described in Fig. 2b.

Multidimensional scaling (MDS) was used as an unsupervised approach to visualize the level of similarity of the 18 RNA samples (Fig. 2c). MDS is a method similar to principal component analysis that enables high level representation of RNA Seq data based on similarities in expression of 500 genes with the largest fold change difference in the data set. In this analysis, distances were calculated as leading log-fold-changes between each sample pair. From Fig. 2c it is readily apparent that MDS differentiates the RNA seq expression patterns of the mesenchymal state from that of the epithelial cells in the first dimension and that of TNFα in the second dimension. Although TNF induces very strong effect on the epithelial cells in the second dimension, the relative response of the mesenchymal cells is highly compressed in the second dimension. Finally, the differences in fold change between replicates in the same group are very small relative to the inter-group responses. These analyses indicated that we had a robust and reproducible dataset and that chronic TGFβ treatment partially appeared to mimic acute stimulation of epithelial cells with TNF.

**Fig. 3:**
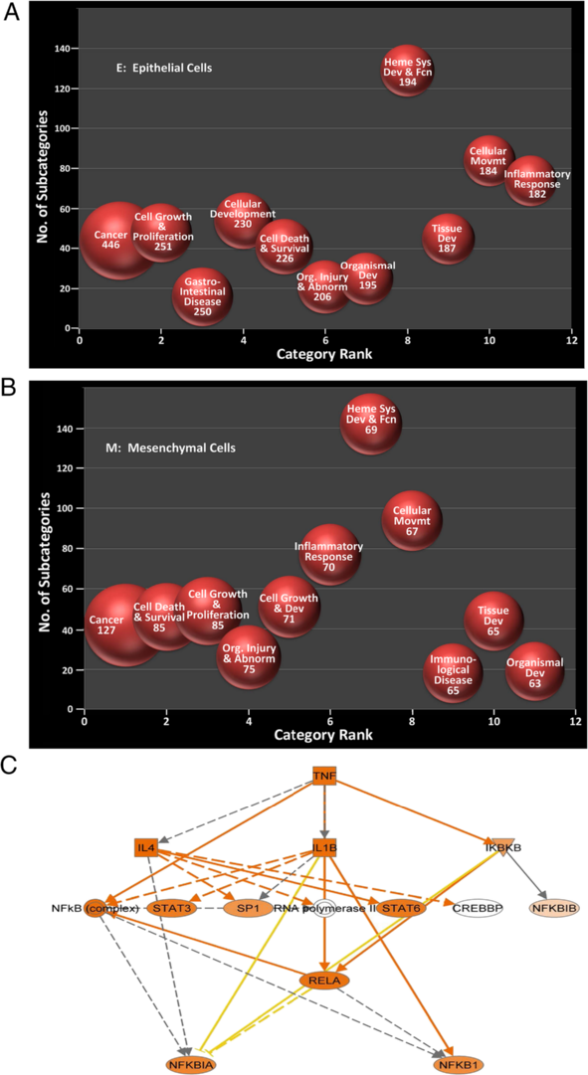
Functional analysis of differentially expressed genes in EMT. **a** Ingenuity pathway analysis (IPA) is shown for genes expressed in control hSAECs. X axis, category rank; Y axis, number of subcategories in each pathway. **b** IPA for genes in hSAEC-EMT. Note the cell death and survival and organismal injury and abnormality become the 2nd and 4th ranked pathway. **c** Enriched TNF signaling pathway identified by IPA in DEG data set. Intensity of red hue is proportional to upregulation by EMT

Generalized linear modeling was used to identify genes that were differentially expressed in the data set; contrasts were used to compare the differences between treatment groups. The results of generalized linear modeling (GLM) are provided in Additional files 2–10. We first focused on the comparison of the mesenchymal *vs* epithelial states. This comparison identified 3,487 differentially expressed genes (DEGs) that met a fold change cut-off of >2 with a highly significant, Bonferroni corrected pvalue (p < 0.00001). This group of DEGs was subjected to Ingenuity Pathway Analysis (IPA), where canonical pathways were ranked by the enrichment of genes in each process. Here we noted the rank ordered appearance of “cell death and survival”, “organismal injury and abnormalities”, and “cellular development” were influenced by the TGFβ-induced EMT (c.f. Fig. 3a, 3b).

Analysis of canonical pathways further showed that NF-κB/RelA-signaling pathway was significantly enriched in a network with SP1 and STATs (Fig. 3c), factors known to modulate RelA-dependent signaling [34, 35].

### Transcription factor enrichment of the EMT

An unbiased transcription factor (TF) enrichment analysis was performed on the DEGs. In this analysis, 345 distinct TF experiments of ChIP-chip, ChIP-Seq, ChIP-PET or DamID were tested for TF enrichment using hypergeometric probability distribution [31]. Here, we only consider the enriched TFs from 148 DNA binding experiments performed on human cells. Of these, 47 distinct TF experiment datasets were significantly enriched for the 3,487 DEGs in EMT. The enriched TFs are shown in Table 4, ranked by enrichment fold ratio and associated with the number of upregulated genes. Many of these TFs are linked to EMT in the literature. Of these, SMADs were among the top highly enriched TFs, consistent with their known role in mediating canonical TGFβ signaling [16]. Also included in the top list was tripartite motif containing 28 (TRIM28), an epigenetic modulator regulating histone H3 modifications on E-cadherin and N-cadherin promoters [36]. SOX2 promotes tumor metastasis by stimulating EMT via regulation of the WNT/β-catenin signal network [37]. ESR1 and ESR2, reported to suppress EMT [38, 39], were also significantly enriched. The CLOCK TF binds the E-box of *PER2* and regulates this and other circadian rhythm genes, which are reported to be involved in EMT [40].

**Table 4:** Significantly enriched human transcription factors for upregulated genes in the EMT process. The enriched transcription factors (TFs) were inferred by comparing ChIP-Seq or ChIP-chip identified target genes (TGs) of each TF with the upregulated DEGs in the EMT process. The enriched TFs were sorted according to the enriched fold ratios

Human TFs	PMID	# total TGs	# DETGs	Fold ratio	Corrected p-value	Cell lines
TRIM28	21343339	85	21	3.9	1.8E-05	HEK293
ESR1	21235772	213	36	2.7	4.8E-04	MCF-7
CLOCK	20551151	398	58	2.3	2.3E-05	293 T
ESR2	21235772	392	57	2.3	9.1E-07	MCF-7
SMAD3	21741376	1074	144	2.1	1.4E-06	HESC
SOX2	20726797	2060	275	2.1	<3.45E-14	SW620
RELA	24523406	1147	152	2.1	<3.45E-14	A549
BACH1	22875853	1183	150	2	<3.45E-14	HELA-AND-SCP4
GATA1	19941826	700	84	1.9	3.8E-14	K562
TP53	18474530	734	88	1.9	4.4E-06	U2OS
GATA2	21666600	634	75	1.9	2.1E-06	HMVEC
PAX3-FKHR	20663909	975	115	1.9	4.8E-05	RHABDOMYOSARCOMA
SMAD4	21741376	2032	237	1.8	2.4E-08	HESC
DROSHA	22980978	441	51	1.8	<3.45E-14	HELA
ZNF217	24962896	1400	160	1.8	8.7E-03	MCF7
NFE2L2	22581777	508	58	1.8	4.7E-11	LYMPHOBLASTOID
EOMES	21245162	861	98	1.8	3.7E-03	HESC
SMAD2	18955504	1643	184	1.8	4.1E-06	HaCaT
SMAD3	18955504	1643	184	1.8	3.8E-12	HaCaT
NR3C1	21868756	1023	111	1.7	3.8E-12	MCF10A
DNAJC2	21179169	803	87	1.7	6.1E-06	NT2
EGR1	20690147	5216	549	1.7	2.4E-04	ERYTHROLEUKEMIA
SOX2	21211035	2830	288	1.6	<3.45E-14	LN229_GBM
GATA2	19941826	1727	175	1.6	<3.45E-14	K562
MYC	19915707	1793	177	1.6	8.5E-08	AK7
AR	19668381	2958	291	1.6	5.0E-07	PC3
ATF3	23680149	1995	193	1.5	8.8E-13	GBM1-GSC
SMAD4	21799915	2302	220	1.5	4.3E-07	A2780
GATA1	21571218	2388	228	1.5	7.2E-08	MEGAKARYOCYTES

*PMID* pubmed article number; *# total TGs* number of ChIP-Seq or *ChIP-chip* identified target genes (TGs) of each TF; *#DETGs* number of differentially expressed genes

The tumor suppressor TP53 is known to regulate EMT [41, 42]. We observed that p53-related TP63, IRF1, TFAP2C, CTNNB1, AHR1, ELF5, FOXM1 and FOXO3 were also exclusively enriched TFs for downregulated DEGs (Table 5). Among these, TP63 attenuates EMT in prostate cells [43] and alternatively spliced isoform ΔNp63α inhibits EMT in human bladder cancer cells [44]. IRF1 is a tumor suppressor modulated by miR-23, promoting TGF-beta-induced EMT in lung cancer [45]. TFAP2C governs the luminal epithelial phenotype in mammary development and carcinogenesis [46]. AHR1 was reported to inhibit TGFβ-induced EMT [47]; ELF5 inhibits Type I EMT in mammary gland development and Type III EMT in breast cancer by transcriptionally repressing SNAIL2 [48]. Constitutive activation of CTNNB1 stabilized mesenchymal phenotypes of epithelial cells [49]. Of relevance to the hypothesis developed in this work, we noted that NF-κB/RelA was among the top significantly enriched TFs, with ChIP-Seq binding sites for 152 of the 1477 upregulated genes in the EMT data set, indicating that NF-κB/RelA dependent genes may play an important role in Type II EMT.

**Table 5:** Significantly enriched human TFs for the downregulated genes in the EMT process. The enriched transcription factors (TFs) were inferred by comparing ChIP-Seq or ChIP-chip identified target genes (TGs) of each TF with the downregulated DEGs in the EMT process. The enriched TFs were sorted according to the enriched fold ratios

Human TFs	PMID	# total TGs	# DETGs	Fold ratio	Corrected *p*-value	Cell lines
TP63	17297297	34	14	3.9	1.1E-04	HaCaT
TRIM28	21343339	85	22	3	7.4E-04	HEK293
ESR2	21235772	392	98	2.9	<3.45E-14	MCF-7
CTNNB1	24651522	138	30	2.5	6.5E-04	LGR5+ INTESTINAL STEM CELL
SOX2	20726797	2060	433	2.4	<<3.45E-141.0E-16	SW620
IRF1	21803131	326	67	2.4	5.9E-09	MONOCYTES
TFAP2C	20629094	1125	229	2.4	<3.45E-14 < 3.45E-14 < 3.45E-14	MCF7
ESR1	21235772	213	43	2.3	4.1E-05	MCF-7
ZNF217	24962896	1400	271	2.2	<3.45E-14	MCF7
FOXM1	23109430	253	47	2.2	1.4E-04	U2OS
NR1H3	23393188	574	104	2.1	8.6E-11	ATHEROSCLEROTIC-FOAM
CDX2	20551321	383	69	2.1	1.2E-06	CACO-2
BACH1	22875853	1183	211	2.1	<3.45E-14	HELA-AND-SCP4
AHR	22903824	621	109	2	1.9E-10	MCF7
FOXO3	23340844	650	114	2	5.9E-11	DLD1
AR	21909140	258	45	2	1.5E-03	LNCAP PROSTATE CANCER CELL LINES
TP53	16413492	276	48	2	7.8E-04	HCT116
SMAD2	18955504	1643	285	2	<3.45E-14	HaCaT
SMAD3	18955504	1643	285	2	<3.45E-14	HaCaT
TP53	18474530	734	122	1.9	3.9E-10	U2OS
RELA	24523406	1147	187	1.9	<3.45E-14	A549
TP63	23658742	3355	541	1.9	<3.45E-14	EP156T
ARNT	22903824	912	147	1.9	2.2E-11	MCF7
SMAD4	19686287	348	56	1.9	1.4E-03	HaCaT
TCF4	18268006	392	63	1.9	3.9E-04	LS174T
CLOCK	20551151	398	62	1.8	1.3E-03	293 T
PAX3-FKHR	20663909	975	144	1.7	3.1E-08	RHABDOMYOSARCOMA
TP63	22573176	3867	570	1.7	<3.45E-14	HFKS
GATA1	19941826	700	103	1.7	1.9E-05	K562
SMAD3	21741376	1074	158	1.7	4.8E-09	HESC
CTNNB1	20460455	900	130	1.7	1.1E-06	HCT116
FOXA2	19822575	2791	402	1.7	<3.45E-14	HepG2
SMAD4	21741376	2032	290	1.7	<3.45E-14	HESC
ELF5	23300383	920	130	1.6	4.2E-06	T47D
GATA2	19941826	1727	241	1.6	5.2E-12	K562
TRIM28	17542650	680	94	1.6	1.2E-03	NTERA2
GATA2	21666600	634	87	1.6	3.6E-03	HMVEC
AR	19668381	2958	398	1.6	<3.45E-14	PC3
HNF4A	19761587	816	108	1.5	1.7E-03	HUMAN INTESTINAL CELL LINE CACO-2
AR	22383394	1690	221	1.5	4.3E-08	PROSTATE_CANCER

*PMID* pubmed article number; *# total TGs* number of ChIP-Seq or *ChIP-chip* identified target genes (TGs) of each TF; *#DETGs* number of differentially expressed genes

The depleted TFs based on the ChEA ChIP-X data set include ELF1, GABP, KDM6A, EST1, ETS1 (Tables 6, 7), suggesting that these TFs may be involved in the reversal of EMT, termed MET. GABP has a predicted binding site on miR200c, an important effector of EMT in immortalized mammary epithelial cells, MCF12A [41]. KDM6A was reported to regulate the expression of TFs critical for stem cell differentiation. EMT is a process of dedifferentiation and gain of stemness, which may explain the depletion of this TF in Type II EMT. EST1 and ETS1 have not yet been reported to be involved in MET. Although our TF enrichment results may be affected by limited experiments in the public databases of human epithelial cells, this analysis has generated valuable hypotheses on the TFs involved in EMT, most of which are supported by the literature.

**Table 6:** Significantly depleted human TFs for the upregulated genes in the EMT process

Human TFs	PMID	# total TGs	# DETGs	Fold ratio	Corrected *p*-value	Cell lines
ELF1	17652178	99	0	Inf	<3.45E-14	JURKAT
GABP	17652178	655	9	4.6	2.2E-08	JURKAT
EST1	17652178	636	9	4.5	6.4E-08	JURKAT
ETS1	20019798	1446	28	3.3	2.8E-14	JURKAT
KDM6A	18722178	410	9	2.9	7.4E-03	U937_AND_SAOS2
FOXP3	21729870	1300	43	1.9	6.1E-05	TREG
VDR	23849224	2029	69	1.9	9.1E-08	CD4+
GABP	19822575	2430	92	1.7	7.2E-07	HepG2
HOXC9	25013753	1858	72	1.6	1.7E-04	NEUROBLASTOMA BE2-C

The depleted transcription factors (TFs) were inferred by comparing ChIP-Seq or ChIP-chip identified target genes (TGs) of each TF with the upregulated DEGs in the EMT process. Depleted TFs were sorted according to the depleted fold ratios

*PMID* pubmed article number; *# total TGs* number of ChIP-Seq or *ChIP-chip* identified target genes (TGs) of each TF; *#DETGs* number of differentially expressed genes

**Table 7:** Significantly depleted human TFs for the downregulated genes in the EMT process. The depleted transcription factors (TFs) were inferred by comparing ChIP-Seq or ChIP-chip identified target genes (TGs) of each TF with the downregulated genes in the EMT process. The depleted TFs were sorted according to the depleted fold ratios

Human TFs	PMID	# total TGs	# DETGs	Fold ratio	corrected *P*-value	Cell Lines
GABP	17652178	655	16	3.5	2.8E-09	JURKAT
KDM6A	18722178	410	14	2.5	2.2E-03	U937_AND_SAOS2
ETS1	20019798	1446	59	2.1	3.3E-10	JURKAT
EST1	17652178	636	28	2	3.3E-03	JURKAT
GABP	19822575	2430	132	1.6	4.4E-08	HepG2

*PMID* pubmed article number; *# total TGs* number of ChIP-Seq or *ChIP-chip* identified target genes (TGs) of each TF; *#DETGs* number of differentially expressed genes

**Table 8:** Gene list in each of the six clusters obtained from hierarchical clustering analysis of 249 genes upon stimulation by TNFα in epithelial and mesenchymal state. Each cluster corresponds to that in Fig. 6b

	Cluster A	Cluster B	Cluster C	Cluster D	Cluster E	Cluster F
1	RNF19B	DUSP6	SNCAIP	PIM3	PLAUR	SLC2A6
2	IRF1	EGR1	TNFAIP2	CLDN1	MAFF	SYNPO
3	GBP1	DUSP5	CSF1	SERPINB1	NKX3-1	TLR2
4	TNFSF15	SLITRK6	DRAM1	STARD5	IL6	COL12A1
5	BTG2	MAT2A	IKBKE	KRT6B	IL8	FEZ1
6	SPRR2D	NAB2	SLC39A8	GBP2	PTX3	PID1
7	HCAR3	CYP1A1		DHRS3	TNF	MGLL
8	HCAR2	FAM110C		SPRR1A	CXCL3	SERPINE2
9	IL1RN	KIAA1551		ITGB8	CXCL2	LBH
10	TNFAIP8	FBXW7		ANKRD33B	NFKBIZ	TNC
11	FOSB	SGK223		PI3	CSF2	MMP9
12	NR4A1	EMP1		CDC42EP4	IL1A	IL7R
13	NCOA7	SPRY2		LYPD3	IL1B	LAMB3
14	SERPINB2	TIPARP		LPAR6	NFKBIA	PIK3IP1
15	ARL5B	SRF		PRSS8	NFKBIE	NAMPT
16	ITPKC	TGIF1		DDX58	NUAK2	ITGA5
17	GATA6	FOSL1		C1orf226	LIF	DNER
18	SDC4	GADD45A		ISG15	TNFAIP3	GPR176
19		RAP2B		CXCL16	TNFAIP6	TGM2
20		OVOL1		SLPI		JPH2
21		PIM1		OAS1		COL27A1
22		HBEGF		TGM1		STC2
23		EREG		MUC1		FSTL3
24		TRIB1		PSTPIP2		SOX4
25		EGR3		SCNN1G		DUSP1
26		PHLDA2		SRD5A1		ST3GAL1
27		MAP3K14		S100A8		FADS3
28		LRIG1		MMP28		BHLHE40
29		KRT15		PRODH		RHOB
30		HAS3		IFIH1		SPRY4
31		IFFO2		ASS1		AKAP12
32		AKR1B10		GCLC		ANGPTL4
33		CKB		LRRC8D		DLC1
34		CALML3		OAS3		THBS1
35		SLC2A1		UBE2L6		SEMA7A
36		AQP3		IFI6		NNMT
37		IL6R		TAP1		ICAM1
38		SESN3		PSMB10		XBP1
39		ZNF462		HLA-F		CXCR7
40		DST		CDKN1C		MSC
41		HR		ALDH2		CXCL1
42		C1orf116		SQRDL		CSF3
43		WEE1		STAP2		ZC3H12A
44		GJB2		PRKAG2		INHBA
45		TSC22D1		SGPP2		SERPINA1
46		CYP1B1		C1R		EDN1
47		BCL10		TRANK1		G0S2
48		ALDH1A3		APOL6		LOC284454
49		PHLDA1		PARP14		BMP2
50		ERRFI1		CXCL10		DKK3
51		TCF4		BIRC3		DKK1
52		STK40		TYMP		SMAD7
53		AP5B1		CD74		PTPRE
54		FAM214A		SERPINA3		F2R
55				OAS2		TNFRSF12A
56				WFDC2		TNFRSF10D
57				SFTPD		ZBED2
58				RHCG		NEDD9
59				MX1		EMP3
60				PSMB9		GFPT2
61				BBOX1		MRAS
62				IFI27		AFAP1L1
63				SLC6A9		IL1R1
64				IFIT3		TLR4
65						MLPH
66						CCBE1
67						IGFL1
68						HRH1
69						AOX1
70						MARCKSL1
71						CHST2
72						SERPINE1
73						KCNJ12
74						MCAM
75						PRSS23

Further comparison of the TF enrichment analysis indicated that the 30 enriched TFs for upregulated DEGs significantly overlapped with the 40 TFs associated with downregulated DEGs (p-value = 4.3e-13), with 18 TFs in common (Tables 4 and 5). A similar finding was observed with depleted TFs in the EMT DEGs (Tables 6 and 7), suggesting that many transcription factors are bimodal, i.e., they both inhibit or activate the target genes depending on the chromatin environment.

### Topographical map of EMT regulated transcription factors and target genes

**Fig. 4:**
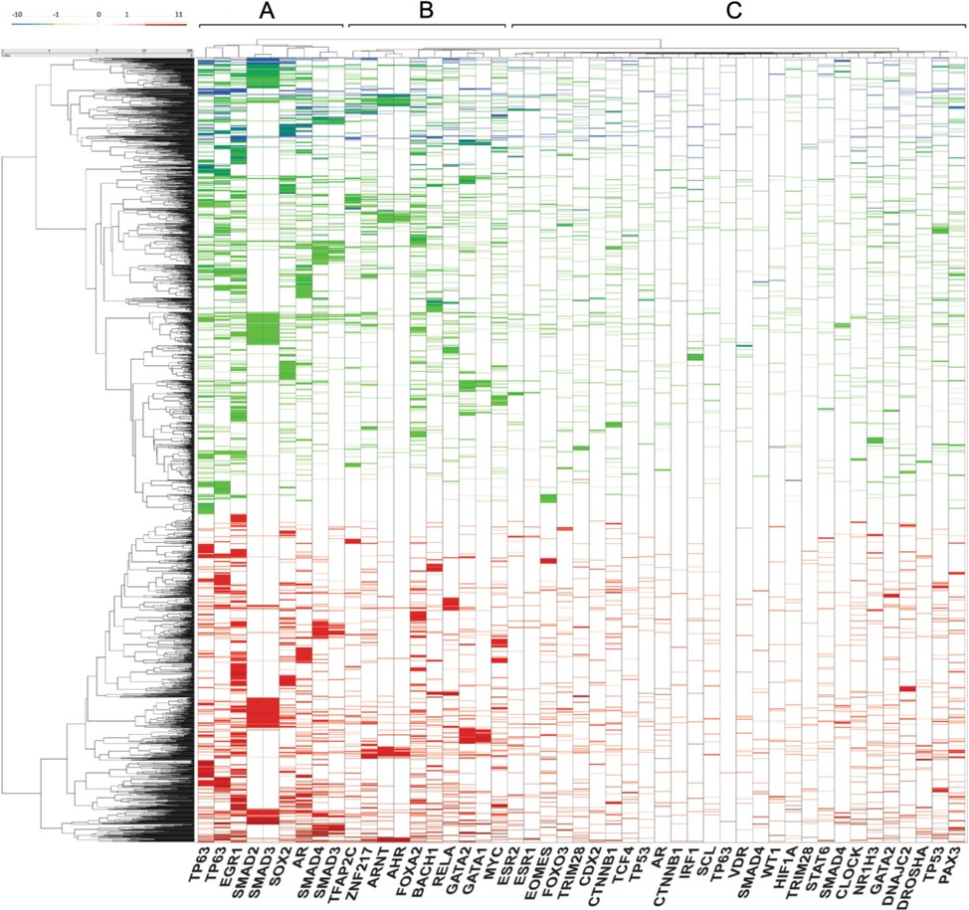
Transcription factor-target gene topological analysis. Hierarchical clustering analysis was conducted on 109 transcription factors (gene names of the enriched transcription factors from 47 experiments are shown in columns) and DEGs associated with EMT using the Euclidean distance measure and the Ward. D2 linkage function. Variance, Skewness, and Kurtosis dendrogram results were compared to 1000 random permutations of the data. Three significant clusters, labeled A, B and C are identified at top. The three clusters were highly significant (*p* < 0.001)

We next sought to map the relationships between TFs and target genes controlling EMT. In this analysis, hierarchical clustering identified three significant TF clusters and two DEG clusters (Fig. 4). The gene clusters correspond to genes that were either up- or downregulated by EMT. Conversely, we noted that each TF is associated with both up- and down-regulated target genes, indicating bimodal behavior consistent with the above TF enrichment analysis. The TF cluster A contains TP63 and Smad2/3. Both Smad 2 and 3 regulate relatively fewer DEGs, some of which are coregulated by EGR and SOX2. TF cluster B contains RelA, BTB and CNC homology 1 (BACH1), globin transcription factor (GATA1/2), and MYC proteins; TF cluster B regulates a distinct group of DEGs from those regulated by cluster A, but also a bimodal fashion. Cluster C is the largest group of TFs containing estrogen receptor (ESR1/2), forkhead box 03 (FOXO3), TRIM28 and others, and is associated with the fewest up- or downregulated DEGs.

### Type II EMT program controls biological functions distinct from than that of Type III EMT

Because Type II EMT occurs in primary epithelial cells without background oncogenic K-ras mutations characteristic of epithelial tumors [50], we hypothesized that Type II EMT induces distinct gene expression profiles. Differences in gene regulation between Type II EMT and Type III EMT may help identify pathways that are specifically involved in fibrosis (Type II EMT) vs invasion and metastases (Type III EMT). We therefore compared the TGFβ-induced gene expression patterns in primary hSAEC with that of an established human epithelial alveolar carcinoma cell, A549. A549 cells are K-Ras activated, keratin positive epithelial cells with features of the lower airways [51, 52]. For this purpose, we compared our Type II model dataset with a publicly available Type III model dataset (GSE177708, NCBI Gene Expression Omnibus). Affymetrix microarrays were used to measure gene expression in Human adenocarcinoma cell line A549 after TGFβ induction of EMT. Genes in the two datasets were matched by Entrez ID, and only genes that were common to both sets, and detected to be expressed in the microarray, were kept for analysis.

**Fig. 5:**
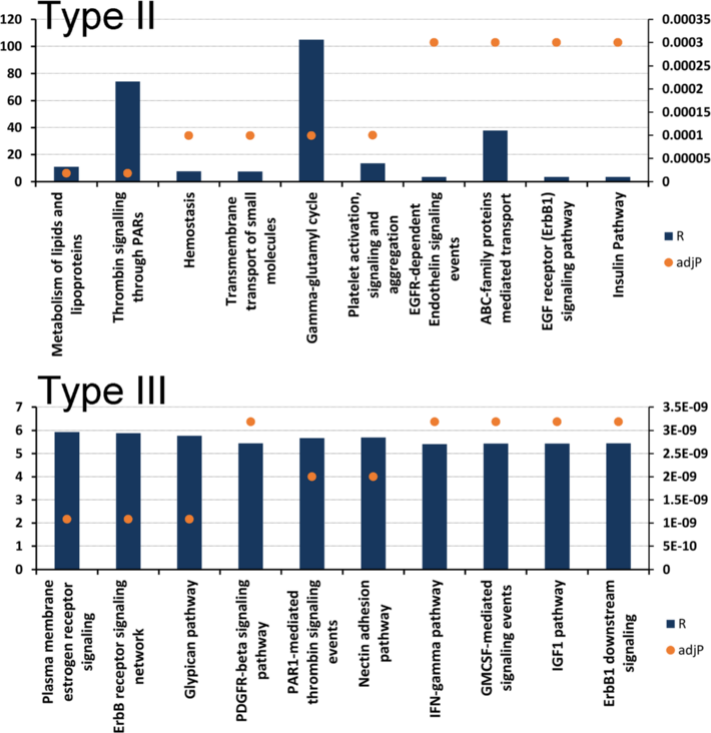
Unique pathways for Type II and Type III EMT. Shown are unique pathways associated with TGFβ-induced Type II EMT (top) and Type III EMT (bottom). Primary y-axis is the Ratio of Enrichment (R) while secondary y-axis is adjusted P-value as calculated by the hypergeometric test. The test is applied to evaluate the significance of enrichment (R), given as p-value (adjP). The value R is calculated by R = k/k_e_, where k = number of genes in our set present in a given pathway, and k_e_ = expected value of k based on the reference set present in the same pathway

We identified 137 genes upregulated in Type II- but not in Type III-EMT, and 124 genes upregulated in Type III- but not in Type II EMT. Fig. 5 (top) illustrates the top 10 canonical pathways for genes uniquely induced in Type II EMT. In this plot, both the p value and ratio of enrichment are presented for each pathway. Here, lipid metabolism and thrombin signaling pathways were the most statistically significant. By contrast, Fig. 5 (bottom) displays the top 10 canonical pathways for genes uniquely induced in Type III EMT. Plasma membrane estrogen receptor signaling, ErbB receptor signaling and glycipan pathways were the most highly significant, consistent with the effect of Type III EMT on cellular motility, invasion and metastasis. Together these data suggest that the induction of Type II EMT produces a cellular biology state distinct from that seen in Type III EMT.

### Modulation of the TNF response by Type II EMT

NF-κB is an inducible transcription factor that is regulated by a rapid release from cytoplasmic IκBα stores, followed by a slower release from 105 kDa NFκB2 complexes referred to as the canonical and noncanonical pathways respectively [53, 54], producing sequential waves of NF-κB dependent gene expression [55, 56]. Our mechanistic studies have found that the noncanonical pathway is coupled to the canonical pathway activation at a fixed time interval. This coupling is due to a feed-forward pathway mediated by inducible expression of the rate-limiting signaling adapter, TRAF1, that triggers activation of the noncanonical pathway [57]. Recently we observed that that the EMT state produces profound changes in canonical-noncanonical pathway coupling through transcriptional reprogramming of TRAF1 and NFκB2 promoters [5, 58]. As a result, the “coupling constant” e.g., the lag time between activation of the canonical and the noncanonical NF-κB program, was markedly reduced by EMT.

**Fig. 6:**
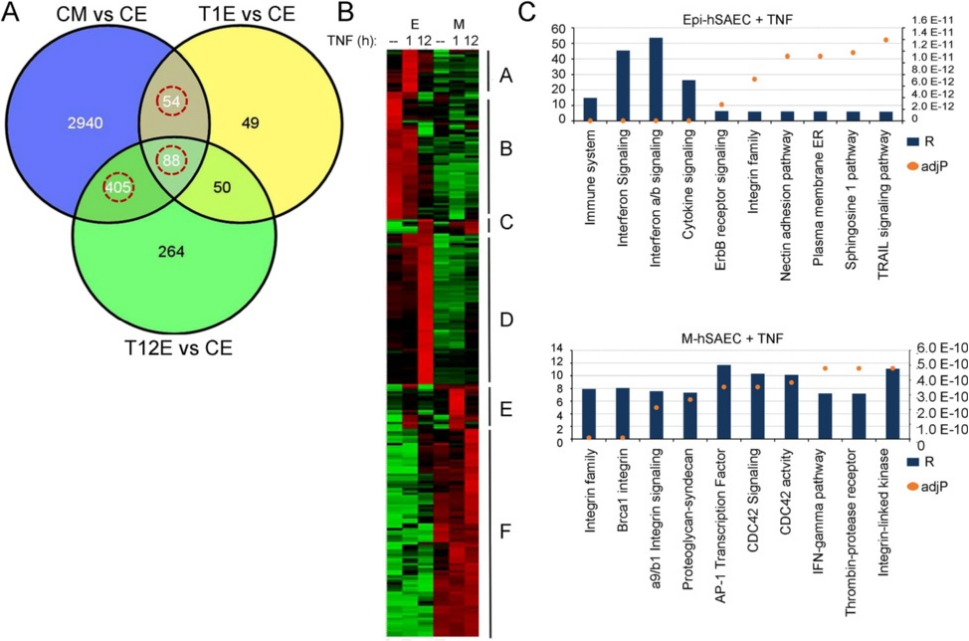
TGF treatment induces a core of TNF regulated genes. **a** Venn diagram of the overlap of TGF regulated genes (CMvsCE) with those induced by TNF at 1 h (T1EvsCE) and 12 h (T12EvsCE) in hSAECs. The overlapping genes between TGF state (CMvsCE) and both or either of TNF 1 h or 12 h were considered for further analysis. This gene set amounts to 547 genes. **b**. Heat map of TNF regulated EMT genes. **a** clustered image heatmap of a normalized matrix was created that correlates gene expression pattern of 249 genes to the time course in HSAECs upon stimulation by TNFα and TGFβ. For each gene, mean and standard deviation were calculated from their expression values across the time course and were normalized to unstimulated HSAECs. Z-score transformation was applied for each gene [5]. Hierarchical clustering was performed using an average-linkage clustering algorithm across the time points in the absence or presence of stimulants. The cluster tree of genes is represented on the y-axis, and time-points and stimulants are shown on the x-axis. Each block of red or green represents a high positive or negative correlation between the gene expression and the stimulant under a specific time point. The list of genes in each cluster are shown in Table 8. (C) Top 10 pathways uniquely associated with TNF-induced genes in epithelial cells (top) and mesenchymal cells (bottom). Primary y-axis is the Ratio of Enrichment (R); secondary axis is adjusted P value (described in Fig. 5)

We therefore hypothesized that TGFβ-induced EMT produced profound differences in the response to activating the TNF signaling pathway. We first compared the TGFβ-regulated gene network with that induced by TNF stimulation. Of the 3,487 DEGs detected in EMT, 547 were also regulated by TNF, indicating that the two signaling pathways are highly overlapping (Fig. 6a). The expression patterns of the TNF-dependent EMT regulators were examined. To reduce noise, the data set was filtered to only the more robustly expressed 249 genes and subjected to hierarchical clustering, resulting in 6 major clusters (A-F, Fig. 6b). Cluster A represented early genes activated in hSAECs and silenced in EMT; Cluster B represented genes downregulated by TNF in hSAECs and not expressed in EMT; Cluster C represented late genes activated in hSAECs and whose expression was potentiated in EMT; Cluster D represented late genes activated in hSAECs and silenced in EMT; Cluster E represented early genes in hSAECs and potentiated by EMT; finally Cluster F represented genes not activated by TNF in hSAECs, but strongly upregulated by TNF in EMT in a late expression pattern. TF enrichment analysis identified 13 shared transcription factors controlling these 6 clusters including androgen receptor (AR), GATA1/2, MYC, NANOG, NR0B1, NR1H3, RelA, SMAD2/3, STAT3, TP-53 and −63 (not shown). These are members from all three of the major TF clusters associated with the EMT program (Fig. 4).

The hierarchical clustering analysis suggested that the TNF pathway elicits distinct biological functions in epithelial cells versus that after Type II EMT. We therefore examined the function of genes activated by TNF in hSAECs but not activated after EMT, and those activated after EMT but not in hSAECs. From this analysis, interferon signaling and immune system activation were prominent in hSAECs (Fig. 6c, top). By contrast, integrin signaling was upregulated by TNF in the EMT background (Fig. 6c, bottom). Integrin αV signaling is particularly important because other studies have demonstrated that integrin signaling drives the maintenance of the EMT state [59], providing an additional cross-talk mechanism for how cytokine signaling reinforces EMT.

Analysis of TNF-dependent genes common to both epithelial and mesenchymal states, revealed that *NFKBIE/I*κ*B*α is found in Cluster E. *NFKBIE/I*κ*B*α is a prototypical early-response gene that we showed earlier was potentiated by EMT. In contrast, *TNFAIP1/Naf1*, a prototypic late gene under noncanonical NF-κB pathway control is found in Cluster F consistent with our earlier observations [5, 55–57]. Together we conclude that TGFβ-induced EMT produces complex modifications of the TNF response program through both potentiation and inhibition of distinct gene subnetworks.

### RelA is essential for TGFβ-induced Type II EMT

Together, the findings that TGFβ induces a gene expression profile similar to that of TNF (Fig. 2), a significant fraction of TGFβ-regulated genes are NF-κB target genes (Table 4), and that TGFβ induces a core of TNF-induced NF-κB-regulated genes (Fig. 6a) led us to investigate the relationship of NF-κB activation with EMT.

**Fig. 7:**
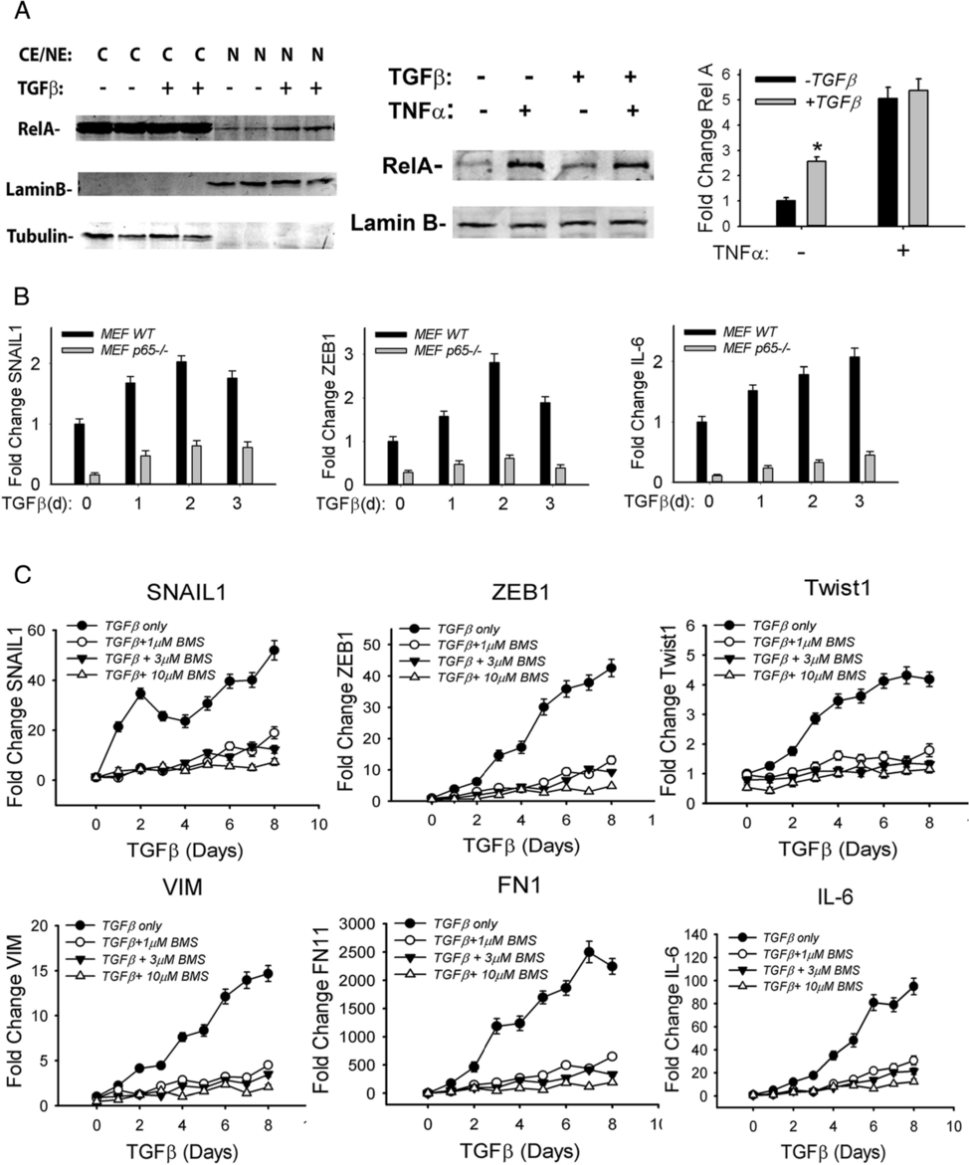
Requirement of NFκB signaling for TGFβ induced Type II EMT. **a** Cytoplasmic and nuclear extracts from hSAECs with or without EMT were isolated. 100 μg of cytoplasmic or nuclear extracts were processed for Western blot using anti-RelA Ab (upper panel). Lamin B and tubulin were detected as loading control Abs respectively. Low left panel: epithelial cells or mesenchymal cells were stimulated with 25 μg/ml TNF for 1 h and processed for RelA Western blot. Lower right panel: Quantification of nuclear RelA from Odyssey infrared imager. * *P* < 0.05 compared with control epithelial cells. **b** Wild-type (WT) or RelA^−/−^ mouse embryonic fibroblasts (MEF) were incubated with TGFβ for 0, 1, 2, 3 days respectively. Total RNA was extracted and expression of EMT core transcription factors (*SNAIL1* and *ZEB1*) and NF-κB-dependent genes (*IL-6*) were measured by Q-RT-PCR. The data shown is from n = 3 independent experiments. **c** Epithelial cells were stimulated with TGFβ for various times in the absence or presence of a small molecule IκB kinase inhibitor (BMS-345541, 1, 3, and 10 μM respectively). The expression of *SNAIL1, Twist1, VIM, ZEB1, FN1* and *IL-6* were measured by Q-RT-PCR. The data shown are from n = 3 experiments

Earlier studies have shown that TGFβ is coupled to NF-κB activation in carcinoma cells by oncogenic transformation induced expression of the TGFβ-associated kinase, TAK1 [60]. Whether TGFβ is coupled to NF-κB activation in primary cells without TAK1 amplification is not known. We therefore measured the relative abundance of nuclear RelA in response to TGFβ-stimulation. We observed a 2.5-fold induction of nuclear RelA 1 h after TGFβ stimulation (Fig. 7a, top panel). We noted that this induction was weaker than the 5-fold induction induced by the prototypical TNFα ligand; together there was no additive effect of the two ligands (Fig. 7a, lower panel).

Two strategies were used to determine the functional requirement of RelA in TGFβ-induced EMT. First, the induction of EMT was examined in RelA^−/−^ mouse embryonic fibroblasts (MEFs). Here, the expression of TGFβ-induced *SNAIL1*, *IL-6* and *ZEB1* were completely blocked in RelA^−/−^ MEFs compared to RelA WT MEFs (Fig. 7b). Second, we measured the effect of IKK inhibition on a time course experiment of TGFβ stimulation in epithelial cells. For this purpose, we used a highly selective, allosteric inhibitor of IKK (BMS-345541); this compound does not affect the JNK, MAPK or Jak-STAT pathways [22]. We observed that TGFβ stimulation produced a complex pattern of *SNAIL1* expression, with a rapid peak of 30-fold induction after 2 days, followed by a decline, with a second peak of 40-fold induction after 5 days of treatment (Fig. 7c). By contrast, in epithelial cells treated with BMS-345541 at concentrations of 1, 3 and 10 μM respectively, *SNAIL1* mRNA expression was significantly reduced at all time points measured. The patterns of *ZEB1* and *Twist1* showed a monotonic increase in expression peaking ~6 days after treatment at 40-fold and 3-fold inductions relative to untreated hSAECs, respectively. Similar to the effect on *SNAIL1*, BMS-345541 significantly reduced *ZEB1* expression and completely blocked *Twist1* expression (Fig. 7c). Similar patterns of inducible expression and BMS inhibition were observed for the mesenchymal cytoskeletal genes, *VIM*, *FN1* and *IL-6* (Fig. 7c). Together these data indicate that IKK-NF-κB/RelA activation is a necessary component of the TGFβ-induced EMT program.

## Discussion

Type II EMT is a dynamic process that mediates airway response to injury, stimulating basal epithelial cells to promote re-epithelialization and extracellular matrix remodeling. EMT transcriptional reprogramming is a coordinated intracellular response triggered by epithelial growth factors (TGF, EGF) signaling via tyrosine kinase-coupled receptor growth factors to converge on a core set of transcription factors including SNAIL1, ZEB1, Twist1, and others. EMT is further modulated by intercellular signaling cross-talk through innate inflammatory cytokines, extracellular matrix and ROS stress. In this study, we employed a well-established model of TGFβ-induced Type II EMT in primary telomerase-immortalized hSAECs to understand the gene expression programs responsible for EMT in normal cells and how they are modified by the innate response. Surprisingly we observe that TGFβ influences a 3,487 member gene network, 547 of which are also controlled by TNF. By comparison to genome-wide ChIP-Seq data sets, we observe that this core pathway is enriched in genes under NF-κB/RelA control. We infer distinct biological functions compared to Type III EMT, demonstrate re-routing of the TNF pathway and experimentally demonstrate the functional role of NF-κB activation in Type II EMT. Finally, we note that the response of the TNF network is markedly affected by the EMT state, with distinct arms of the response being silenced, while others are enhanced. These studies are the first to our knowledge that demonstrate the unique TGFβ signatures in inducing Type II EMT and the requirement of NF-κB in this process.

### TGFβ-induced Type II EMT

TGFβ activates cells by TGFβ receptor type II (TGFβRII), a transmembrane serine- threonine kinase that recruits and phosphorylates TGFβRI on the cell membrane [7]. Activated TGFβRI triggers two inter-related pathways, known as the canonical (SMAD-dependent) and noncanonical (ERK/MAPK-dependent) pathways, that converge on a core set of transcription factors responsible for coordinated suppression of epithelial genes and induction of mesenchymal genes. In the canonical pathway, the activated TGFβRI/II complex phosphorylates cytoplasmic Smads 2/3 that bind to Smad4. This trimeric Smad complex binds to regulatory sequences in the E cadherin promoter, leading its repression and subsequent loss of adherens junctions [12]. In our study, we have found that SNAIL1 expression is the most rapidly induced member of the core EMT regulators (Figs. 1b and 7c). Based on its rapid appearance, we suggest that SNAIL1 expression is one of the early triggers of Type II EMT. The absence of SNAIL1 in the public ChIP-Seq datasets prevented its enrichment and target gene analysis.

Other work has shown that the activated Smad 2/3/4 trimer also binds to Smad-binding elements in the regulatory regions of *JunB* and *c-Jun* genes, inducing their interaction with other coactivators including the EP300 coactivator [9]. This result indicates that the activities of the EMT core TFs are modulated by the EMT state, producing a bi-modal behavior, both activating and inhibiting gene expression. To this end, we note that over half of the TFs identified by enrichment analysis associated with upregulated DEGs in EMT are also associated with the downregulated genes in EMT. We note that these TFs include Smad2/3, RelA, SOX2 and others. Of relevance here, our earlier inference of RelA modulator-target gene triplets showed 6 patterns of modulator interaction with RelA involved in activation and inhibition of specific target genes within biologically relevant pathways [35]. In extension of these studies, we predicted that *FN1* and *FOXP1* function as modulators of RelA action in the setting of EMT [61]. These latter predictions suggest that the target genes and behavior of RelA are influenced by the EMT. It will be of interest to develop systems-wide maps of the effects of TGFβ on expression of modulators for the NFκB/RelA and other EMT-regulated TFs.

Activated TGFβRI complex triggers a Smad-independent (“noncanonical”) pathway through the PI3K/Akt, Ras small GTPases, Wnt/β-catenin, ERK, p38, and JNK kinases [7]. Although the actions of Smads are dominant on EMT, signaling through the noncanonical pathway is required for the full expression of EMT. Recent findings in non-small cell lung cancer cell lines indicate that phospho-Erk1/2 mediates a decrease in *ECad* and an increase in *FN1* expression [62]. Also, inhibition of JNK, p38 and Akt activities without affecting Smad phosphorylation blocks TGFβ1 induced α*-SMA*, *SNAIL1* and *col1A* in primary alveolar epithelial cells [63]. Together, these data indicate that Smad signaling is necessary but not sufficient for EMT. In the TF-TG topology map of EMT (Fig. 4), we note that the core TFs are clustered into three major clusters. We suggest that the TGs associated with Cluster A, containing Smad 2/3/4, TP63 and SOX2, are genes downstream of the canonical TGFβ signaling pathway.

### Transcription factors controlling Type II EMT

We computed the over-representation of 148 TF targets from publically available ChIP-chip, ChIP-Seq, ChIP-PET or DamID data [38] using a hypergeometric probability distribution to tentatively identify regulators of the DEGs in EMT. 18 of the 30 enriched transcription factors in the EMT upregulated genes overlapped with the 40 transcription factors regulating the EMT downregulated genes (*p-value* = 4.3e-13), indicating that these 18 had bimodal activities in a target gene dependent manner. Many of have been associated with EMT. In addition to Smad and TP63 proteins discussed above, NF-E2-related factor-2 (NFEL2l2/Nrf2) is negatively regulated by E-cadherin expression, whose downregulation may promote Nrf2 nuclear translocation, resulting in the enhanced resistance of transformed cells to ROS stress [64]. The transcriptional repressor BACH1 has been shown to be upregulated in metastable Type III EMT [65]. We note that *RelA* and *BACH1*, *GATA1*, *GATA2* and *TFAP2C* are enriched in the upregulated genes, whereas only *TFAP2C* is enriched in the downregulated gene set. This observation suggests to us that the activation mode of RelA is modulated by association of distinct coactivators, discussed below. Further dissection of this transcription network will be of interest. For example, the implication of the histone acetyltransferase, CLOCK, in the EMT program and depletion of KDM6A have not been reported to our knowledge and merits further investigation.

### TNF and TGFβ cross-talk

TGFβ-induced EMT is modulated by a variety of signals including cytokine stimulation, morphogen signaling, and ECM interactions. Of relevance here, studies of transformed alveolar basal epithelial cells show that TGFβ-induced EMT is accelerated by the presence of members of the proinflammatory TNF/ IL-1 superfamily of cytokines [16, 17]. TNF/ IL-1 signal through highly conserved death domain containing receptors activating downstream Ras GTPase, p38 MAPK and JNK pathways, shared components of the noncanonical TGFβ signaling pathway [7, 58]. By contrast to this modulatory role our observations in normal epithelial cells indicate that the TNF signature is a major component of TGFβ-induced EMT (Fig. 5a). Pathway analysis of the DEGs in EMT indicates enrichment of TNFR signaling pathways (Fig. 3d) and we observe statistically significant enrichment of RelA target genes mapping to ChIP-Seq datasets (Table 4).

### Requirement of NF-κB in Type II EMT

NF-κB is a master regulator of airway inflammation and cell fate determination. Our previous genome-wide analysis using whole genome-wide RNA-Seq and ChIP-Seq approaches have shown that this transcription factor regulates a ~4,000 member gene network mediating anti-apoptosis, inflammation, and adaptive immunity [34, 66]. In studies of cancer-associated Type III EMT, NF-κB is required for IGF-induced EMT by directly inducing *SNAIL1* [67]. NF-κB has also been shown to upregulate *ZEB1/2* and *Twist1* [20], explaining, in part, how the IL-1/TNF superfamily of cytokines mediates Type III EMT. Our studies indicate that SNAIL1 is the earliest upregulated core transcription factor in Type II EMT and whose significant induction precedes that of ZEB1 and Twist1, leading us to suggest that SNAIL1 is an initial trigger of EMT.

Our study here shows that chronic TGFβ stimulation induces nuclear translocation of NF-κB/RelA. Other studies have shown that TGFβ is a potent activator of NF-κB/RelA in Ras-transformed cell lines. However, the relevance of these findings to normal epithelial cells is confounded by the observations that K-ras is a potent activator of NF-κB [68], and that oncogenic transformation induces expression of the TGFβ-associated kinase, TAK1, coupling NF-κB to TGFβRI [60]. Our western blot quantification indicates that TGFβ treatment in primary epithelial cells weakly induces NF-κB/RelA translocation at levels significantly less than the prototypical TNF monokine (Fig. 7a).

NF-κB/RelA translocation is controlled by two distinct pathways controlling sequential waves of NF-κB dependent gene expression [55, 56]. The canonical pathway liberates cytoplasmic NF-κB/RelA from IκBα complexes in response to IKKα∙IKKβ [54], whereas the noncanonical cross-talk pathway liberates cytoplasmic NF-κB/RelA from NFκB2 complexes in response to IKKα∙NIK. Our experiments using various doses of the allosteric IKK inhibitor indicate that expression of the core EMT genes is significantly inhibited at very low concentrations of BMS-345541. At this 1 μM concentration, BMS is relatively selective for IKK, over that of other related MAPKs [22]. This behavior, together with our observations that RelA knockout cells are also defective in TGFβ-induced SNAI1 and ZEB expression, support our conclusion that the IKK-NFκB pathway is required for type II EMT. More work will be required to identify whether the NF-κB pathway activated by TGFβ is via the canonical, noncanonical or a combination of the two pathways.

### EMT induced alterations in the TNF signaling program

Our earlier genome-wide microarray studies discovered that tonic cellular stimulation by TNF produces sequential waves of NF-κB-dependent gene expression, with each wave controlling distinct biological functions [58, 59]. The most rapidly inducible, “early” genes were a group encoding cytokines, important in paracrine signaling, whereas late genes were those involved in antigen processing and cell surface presentation of MHCI complexes [56]. Mechanistically, the early genes are under canonical pathway control, and the late genes by noncanonical pathways [55, 56]. Recently we observed that that the EMT state produces both profound accentuation of early gene expression and changes in canonical-noncanonical pathway coupling resulting in shortening the coupling interval between them [5, 58]. Although the mechanisms are still under investigation, part of this effect was through transcriptional reprogramming of NFκB-dependent promoters, including that of *TRAF1* and *NF*κ*B2* [5, 58].

In this study we observe that the EMT state markedly changes the TNF program in unexpected ways. Although a cluster of early and late genes (Clusters E, C, respectively Fig. 6b) are potentiated by EMT, our data suggest that this is not a uniform behavior. Interestingly, large subgroups of early and late genes (Clusters A, D, Fig. 6b) are largely silenced in the EMT state. Conversely, a large cluster of genes are activated in a late gene expression pattern in mesenchymal cells that is not activated in normal epithelial cells (Cluster F, Fig. 6b). Pathway analysis shows that the TNF program in normal hSAECs is coupled to interferon signaling and innate immunity. By contrast, the TNF program is re-routed by Type II EMT to elicit the integrin signaling program, an essential pathway controlling maintenance of EMT [59].

How might the TNF signaling pathway be affected so dramatically by EMT? One explanation for this complex behavior is through contextual effects of NF-κB associated modulators. In a recent study, we conducted a probabilistic inference of modulator activity on RelA activity using experimentally determined protein-protein interactions integrated with ChIP-Seq and a compendium of microarray data. The action identified 8349 modulator-RelA-target gene triplets, whose target genes were regulated in six distinct expression modes [35]. Using this approach, a preliminary inference of EMT-regulated NF-κB/RelA modulators identified *FOXP1*, a tumor suppressor gene involved in cardiac development; *FN1* and *CCDC80*, interacting gene involved in extracellular matrix formation; *ACTN1*, a gene important in formation of actin polymers; and *DOCK10*, a Rho GTPase associated with cytoskeletal motility, as key modulators of the NF-κB pathway in EMT [61]. These functions may suggest how the EMT state may modulate the activity of the ubiquitous NF-κB/RelA in a cell-state dependent manner. More investigation will be required to determine the effect of EMT on the expression of RelA modulators. Moreover, these data suggest that the biological consequences of activating the TNF program are distinct in the normal vs EMT state.

### Differences in Type II vs III EMT gene expression programs

TGFβ-induced Type II EMT leads to disruption of mucosal barrier function by inducing the loss of apical polarity, reduced epithelial cadherin and disruption of epithelial adherens junctions [6], express α-SMA stress fibers and intermediate filament VIM, to produce ECM through secretion of Col1A and FN1, and to increase expression of MMPs to promote airway remodeling. This cellular biology is a distinct biological phenomenon from that of Type III induced in a primary transformed cell background, associated with acquisition of motility and resistance to chemotherapeutics.

## Conclusions

Analysis of the gene program triggered by TGFβ has led to a new understanding of the Type II EMT process. First, we have identified a core set of TNF-inducible genes enriched in experimentally determined NF-κB/RelA binding sites. Second, we demonstrate that activation of NF-κB/RelA is required for initiation of TGFβ-induced core EMT TFs and mesenchymal genes. Third, we provide evidence that the gene program induced by EMT in normal epithelial cells confers distinct biological properties than that induced in mesenchymal cells. Finally, we demonstrate that the evolution of the TNF pathway is distinct in the EMT state, altering innate immunity and reinforcing EMT via integrin αV signaling. These data have implications for the effect of EMT on airway mucosal response in chronic respiratory disease.

### Availability of supporting data

The RNA-Seq sequence data have been deposited in the NCBI GEO (Gene Expression Omnibus) database under the accession number GSE61220.

## Additional files

Additional file 1:
**QA/QC of RNA Seq.**
Additional file 2:
**Result of GLM for comparison of DEGs in E vs M state (no TGFβ treatment vs plus TGFβ).**
Additional file 3:
**Result of GLM for comparison of DEGs in E state: 1 h TNF vs Control.**
Additional file 4:
**Result of GLM for comparison of DEGs in E state 12 h TNF vs Control.**
Additional file 5:
**Result of GLM for comparison of DEGs in E state 12 h TNF vs 1 h TNF.**
Additional file 6:
**Result of GLM for comparison of DEGs after 1 h TNF E state vs 1 h TNF M state.**
Additional file 7:
**Result of GLM for comparison of DEGs in 12 h TNF E state vs 12 h TNF M state.**
Additional file 8:
**Result of GLM for comparison of DEGs in M state: 1 h TNF vs Control.**
Additional file 9:
**Result of GLM for comparison of DEGs in M state 12 h TNF vs Control.**
Additional file 10:
**Result of GLM for comparison of DEGs in M state 12 h TNF vs 1 h TNF.**
